# Histological Evaluation of Restylane Lyft Used as a Scaffold for Dental Pulp Regeneration in Non-Infected Immature Teeth in Dogs

**DOI:** 10.3390/ma15124095

**Published:** 2022-06-09

**Authors:** Norah A. AlHowaish, Dina I. AlSudani, Rita Khounganian, Nehal AlMuraikhi

**Affiliations:** 1 Department of Restorative Dental Sciences, College of Dentistry, King Saud University, Riyadh 11612, Saudi Arabia; norah.alhowaish@gmail.com; 2 Department of Oral Medicine and Diagnostic Sciences, College of Dentistry, King Saud University, Riyadh 11545, Saudi Arabia; ritak@ksu.edu.sa; 3 Stem Cell Unit, Department of Anatomy, College of Medicine, King Saud University, Riyadh 11461, Saudi Arabia; nalmuraikhi@ksu.edu.sa

**Keywords:** regenerative endodontic procedures, tissue engineering, tissue regeneration, hyaluronic acid, Restylane, scaffolds

## Abstract

Commercially available hyaluronic acid dermal fillers used as a scaffold in regenerative endodontic procedures (REPs) have demonstrated attractive potentials. This study aimed to histologically evaluate the outcome of REPs using Restylane Lyft (HA) as a scaffold. REPs were performed on pulpless, immature roots in dogs (*n* = 69). The roots were divided into four groups: blood clot (BC), Restylane Lyft (BC + HA), negative control, and positive control. At 13 weeks postoperatively, hard tissue formation, vascularization, the presence of vascularized soft connective tissue and collagen fibers, the degree of inflammation within pulp spaces and/or periapical tissues, and apical closure were evaluated histologically. The vascularization and formation of loosely arranged collagen fibers within the regenerated soft connective tissues were observed significantly more in the BC+HA group (85% and 40%, respectively; *p* < 0.05) compared to the BC group (54.6% and 9.1%, respectively; *p* < 0.05). The degree of inflammation was significantly higher in the HA group than in the BC group; moderate to severe inflammatory cell infiltration was seen in 45% and 13.6% of the cases, respectively. The results of the present study suggest that Restylane Lyft combined with a blood clot used as a scaffold may improve the outcomes of REPs in non-infected, pulpless, immature teeth in dogs.

## 1. Introduction

There have been many advancements in regenerative endodontic procedures (REPs) since the earlier clinical attempts related to pulp regeneration were reported [1,2,3]. REPs are biologically based procedures that allow the cessation of disease signs and symptoms, leading to apical periodontitis healing, root development continuation, the thickening of the root canal walls, and the apical closure of roots of immature, permanent teeth [4]. The achievement of favorable outcomes for REPs primarily relies on the interaction between the three critical elements of tissue engineering: stem cells, scaffolds, and growth factors [5].

Scaffolds are essential elements in pulpal regeneration. An ideal scaffold should possess several physical and biological properties, such as dimensional stability, sufficient porosity with adequate particle size, and biodegradability at a rate similar to that of new tissue formation [6]. Ideally, a scaffold should be a 3D biomaterial that supports the spatial growth of stem cells into the pulp space and regulates the interaction of stem cells with biological molecules in the 3D microenvironment [7]. Endogenous scaffolds, such as blood clots (BCs), platelet-rich plasma (PRP), and platelet-rich fibrin (PRF), have been used in REPs with positive clinical outcomes [1,3,8,9]. However, these scaffolds exhibit several limitations. It has been hypothesized that BCs are relatively unstable and could undergo a quick breakdown, leaving an empty root canal space, thus resulting in the loss of tissue support in the initial phases of the regeneration process [10]. In addition, the induction of bleeding from periapical tissues is not always achievable [11]. Other endogenous scaffolds, such as PRP and PRF, require complex, invasive procedures, including blood withdrawal from the patient, the preparation of special equipment, and staff training. Several alternative exogenous scaffolds have been evaluated for their use in REPs, including natural and synthetic materials, such as polymers [12,13,14], hydrogels [15,16,17], and nanomaterials [18].

Commercially available, injectable, synthetic polymer-based materials are broadly used in cosmetic medicine, and they were initially used for orofacial soft tissue augmentation. Hyaluronic acid (HA)-based hydrogel is a natural polymer present in several soft connective tissues of the human body. As it possesses unique chemical and biological properties, it has been used to construct many synthetic HA-based hydrogels that can be designed with specific properties to be used for different purposes [19]. Moreover, HA-based hydrogels have been introduced as potential scaffolds in several fields of tissue engineering, including dental pulp regeneration.

Hyaluronic acid (HA) scaffolds have been evaluated in a few in vitro investigations regarding dental pulp regeneration. They have shown promising capabilities in promoting the mineralization and differentiation of dental pulp stem cells (DPSCs) [20,21,22,23]. Most commercially available HA dermal fillers share similar features. The mechanical integrity of these materials was greatly enhanced via crosslinking technology due to their hydrogel structure. Moreover, they are commercially available and involve controlled preparations that have been approved by the Food and Drug Administration (FDA) [24]. They can be easily injected as scaffolds into root canals in REPs. HA-based hydrogel has been demonstrated to have promising potential in promoting cell viability, mineralization, and differentiation into odontoblastic-like cells [25,26]. 

Restylane Lyft is an HA-based sterile hydrogel that is FDA approved and commercially available as an injectable dermal/subdermal filler. Previous studies have shown promising results with regard to Restylane and Restylane Lyft when used as a scaffold for REPs in vitro [25,26]. However, to the best of our knowledge, to date, no study has investigated the performance of a Restylane Lyft scaffold in REPs in vivo. Therefore, this study aimed to histologically evaluate the regenerative potential of Restylane Lyft combined with the blood clot when used as a scaffold in REPs in dogs’ immature teeth. The tested null hypothesis states that there are no statistically significant differences between the BC and the Restylane-Lyft-combined-with-the-BC scaffolds in the histological outcomes of REPs, resulting in similar pulpal and periapical responses regardless of the scaffold tested. 

## 2. Materials and Methods

This study was performed in conformity with the NIH guidelines for the care and use of laboratory animals [27]. Experimental procedures were carried out in the Prince Naif Bin Abdulaziz Health Research Center, Experimental Surgery and Animal Lab, King Khaled University Hospital, Riyadh, Saudi Arabia.

### 2.1. Sample Size Calculation and Allocation into Study Groups

The sample size was calculated using G*Power 3.1.9.4 software (Germany) based on a previous study [28]. At a significance level (α) = 0.05, an estimated (SD) = 0.6, an estimated effect size = 0.5, and power = 0.92, it was determined that the sample size should have been at least 20 for each group. Considering a 20% attrition rate, the corrected sample size for each group = 24. Sixty-nine immature roots of upper and lower premolar teeth in three sighthound mixed-breed dogs who were approximately 6 months old were included (24 lower premolars and 18 upper premolars). A block randomization design was applied to assign teeth into the blood clot group (BC), the Restylane Lyft group (BC + HA), the negative control group (NC), and the positive control group (PC).

### 2.2. Surgical Procedure

#### 2.2.1. Animals’ Anesthesia

All surgical procedures were performed under general anesthesia with an intramuscular administration of 8–10 mg/kg Ketamine (Ketaset, Fort Dodge, IA, USA) and 0.02–0.04 mg/kg Atropine (Atroject SA, Henry Schein Animal Health, Portland, ME, USA), followed by an intravenous injection of Xylazine (Sedazine, Fort Dodge, IA, USA) (2.2 mg/kg). Before any intervention, preoperative periapical radiographs (7.0 mA, 60 kVp, 0.66 s) were taken for the included teeth to confirm incomplete root formation and open apices and to record an estimated working length for each root. Each root was considered an individual unit, and roots of the same tooth were allocated to the same group. All surgical procedures were performed by one operator with microscope magnification (System Contraves & Sec, Carl Zeiss, Thornwood, NY, USA). The included teeth (except for the positive control) were anesthetized via local infiltration with 2% Xylocaine without adrenaline (Dentsply Ltd., Surrey, UK). All groups were included in each quadrant of all animals. The treatment procedures were accordingly performed for each study group following the methodology described previously by other investigators [28] as follows:

##### Blood Clot Group (BC) (*n* = 24 Roots)

After single-tooth isolation using a rubber dam, the operative field was disinfected with 10% iodopovidone solution. The access cavity was prepared with a sterile size 2 round tungsten bur (Brassler, Savannah, GA, USA) mounted on a high-speed handpiece (Dentsply, York, PA, USA) with water coolant. Pulp tissues were removed with a #35 K-file introduced to the working length, established 1 mm from the radiographic apex. Special care was taken to avoid touching the canal walls. The canals were then irrigated with 1.5% NaOCl (20 mL/canal, 5 min), followed by 17% EDTA (20 mL/canal, 5 min) using a side-vent irrigation needle placed 1 mm short of the working length. The canals were finally dried with sterile absorbent paper points (Dentsply Maillefer, Ballaigues, Switzerland).

Intracanal bleeding was evoked by gentle over-instrumentation with a sterile size 30 K-file beyond the root apex until the canal was filled with blood to the level of the cementoenamel junction (CEJ). Then, the clot was gently pressed using a sterile cotton pellet soaked in saline and placed 1–2 mm apical to the CEJ to stabilize the BC. A 2–3 mm white ProRoot MTA (DENTSPLY International, Inc., Tulsa, OK, USA) plug, mixed according to the manufacturer’s instructions, was then placed on top of the blood clot. The teeth were restored with light-cured glass ionomer cement (GC Fuji II LC Capsule, GC Corporation, Tokyo, Japan) and kept out of occlusion.

##### Restylane Lyft Group (BC + HA) (*n* = 24 Roots)

Tooth isolation and pulp space disinfection were performed similarly to the BC group. Intracanal bleeding was evoked similarly to the BC group, except that the bleeding was maintained to a level 1 mm below the CEJ. Subsequently, approximately 0.1 mL of Restylane Lyft was carefully injected into the root canals and mixed with the blood using a size 30 K-file until it was homogeneously distributed within the blood. After that, the restorative procedure was carried out similarly to the BC group.

##### Negative Control Group (NC) (*n* = 10 Roots)

Tooth isolation and access cavity preparation were performed similarly to those for the experimental groups. The dental pulps were mechanically disrupted, and all the pulpal tissues were completely removed under copious irrigation with 1.5% NaOCl (20 mL/canal) followed by irrigation with sterile saline (20 mL/canal). The access openings were then restored with a sterile cotton pellet and GIC. The teeth received no further treatment until the end of the study. The roots in this group were used to simulate the clinical situation of discontinued root development in immature teeth due to the loss of pulp vitality, to act as a reference of these teeth when left without treatment, and to demonstrate the positive effects of the REP in the experimental groups.

##### Positive Control Group (PC) (*n* = 11 Roots)

This group included healthy teeth of the same type as those in the previous groups, kept without any intervention to serve as a radiographic and histologic reference for normal physiological root development.

##### Animal Postoperative Care

Animals were closely monitored for any signs of discomfort or changes in eating habits. Plaque control procedures were performed twice weekly via the topical application of 0.2% chlorhexidine digluconate solution using a toothbrush (GUM, SUNSTAR, Suisse S.A., Etoy, Switzerland).

##### Animal Euthanization and Jaw Resection

At 13 weeks postoperatively, the animals were sacrificed under general anesthesia with 30 mg/kg intravenous socumb (pentobarbital; Butler Company, Columbus, OH, USA). The carotid arteries were exposed and canulated. The animals were euthanized with additional pentobarbital (Socumb, Butler Company, Columbus, OH, USA) at a 90 mg/kg intravenous dose. For tissue fixation, the animals were perfused with 10% buffered formalin (Fisher Scientific, Fair Lawn, NJ, USA) [29]. The jaws with the involved teeth were resected using an electric saw (Leica SP 1600, Deer Park, IL, USA) and post fixed with 10% neutral-buffered formalin solution for 3 days.

### 2.3. Micro-Computed Tomographic Scanning (µ-CT)

To confirm apical closure, µ-CT was used. Each sample was performed by individually securing it with paraffin wax film and mounting it in a polyethylene plastic container. Samples were then positioned on the micro-stage inside the specimen chamber. Scanning was acquired using the Bruker SkyScan 1172, 50 mm FOV, high-resolution micro-CT (Bruker SkyScan, Kontich, Belgium). The scanner configuration used was 93 kV voltage, 106 µA anode current, 158 ms exposure time, 25 µm image pixel size, Al 0.5 mm, 0.3 rotation step for 360° angle, frame averaging of 4 for improved signal-to-noise ratio, and random movement of 8 minimize ring artifacts. A flat-field correction was performed before the scanning procedure to correct variations in the camera pixel sensitivity. After the scanning, a reconstruction of the projected images was performed using ©N-Recon, program version 1.6.9.4 (Bruker SkyScan, Kontich, Belgium) to produce a reconstructed cross-section image. Numerical parameters needed to establish the best image results were checked and adjusted. A ring artifact reduction of 5 for non-uniformity of the background image taken by the X-ray camera; 25% beam hardening compensation to prevent the specimen from appearing artificially denser at or near its surface, and less dense at its central parts; and a smoothing of 2 using Gaussian kernel were applied. A 16-bit TIF file format was the choice selected for saving the images because of the variety of densities comprising the specimen. Reconstructed images were loaded and viewed in Slicer Software (http://www.slicer.org, accessed on 1 February 2022) [30].

### 2.4. Histological Preparation

Immediately after µ-CT scanning, the excised jaw segments were decalcified in a solution containing equal parts of 50% formic acid and 20% sodium citrate for 12 weeks; the solution was changed twice a week. Following decalcification, the jaw blocks were longitudinally cut with each individual root, including the surrounding periapical bone and tissues. The specimens were processed according to the conventional pathological protocols and stained with Hematoxylin and Eosin (H&E) and Masson Trichrome (MT) (Baso Diagnostic Inc., Zhuhai, Guangdong, China) and assessed under a light microscope (Leitz Laborlux 11 Leica Microsystems GmbH (Wetzlar, Germany).

### 2.5. Histological Evaluation

Two trained evaluators assessed the histological slides after a training session regarding the evaluation criteria. They graded them for the following parameters: (1) the presence or absence of new hard tissue on the dentinal root walls, (2) the type of hard tissues formed within the pulp space, (3) vascularization within the pulp space, (4) the presence or absence of soft connective tissue with fibroblasts and collagen fibers within the pulp space, (5) the degree of inflammatory cells that infiltrated within the pulpal space and/or in the periapical area, and (6) the presence or absence of histological apical closure. The two evaluators simultaneously examined the same slide blindly and independently using a multiheaded microscope. The kappa statistic values for inter-examiner reliability were ≥0.85 for all the parameters, indicating a good agreement between the two evaluators. In the case of disagreement, a discussion was undertaken until a consensus was reached. The intracanal and periapical tissues were described based on the histological evaluation criteria. They were scored based on a modification applied from previous histological evaluation criteria brought forward by other investigators [31,32], as defined in Table 1.

### 2.6. Statistical Analysis

Statistical analysis was conducted using the SPSS software (version 23.0, SPSS IBM, Armonk, NY, USA). Chi-square and Fisher’s exact tests were used to compare differences between the groups for the categorical histologic parameters. The Mann–Whitney U test was used to analyze differences in the levels of inflammation between groups. The level of significance was set at *p* < 0.05.

## 3. Results

**Figure 1:**
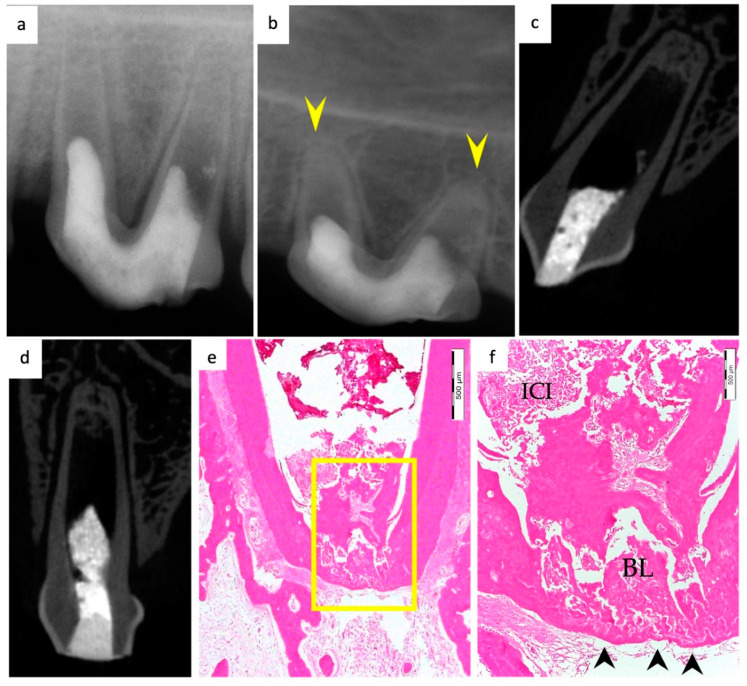
Representative radiographic, µ-CT, and histological photomicrographs of root samples from the BC group. (**a**) Immediate postoperative periapical radiograph showing immature roots and (**b**) 13-week postoperative periapical radiograph showing apical closure in both roots (yellow arrowheads). (**c**,**d**) Thirteen-week postoperative µ-CT coronal views of the distal and mesial roots, respectively, with evident apical closure and mineralized tissue deposition apically. (**e**) Histological evidence of scattered bone-like hard tissue deposition within the root canal in the apical region surrounding connective tissue spaces with apparent apical closure (H&Ex200). (**f**) A magnified, detailed view of the yellow-boxed region in (**e**), showing new bone-like hard tissue formation (BL) in the apical region, causing apical closure (black arrowheads), and mild inflammation (ICI) is evident within canal space (H&Ex400).

**Figure 2:**
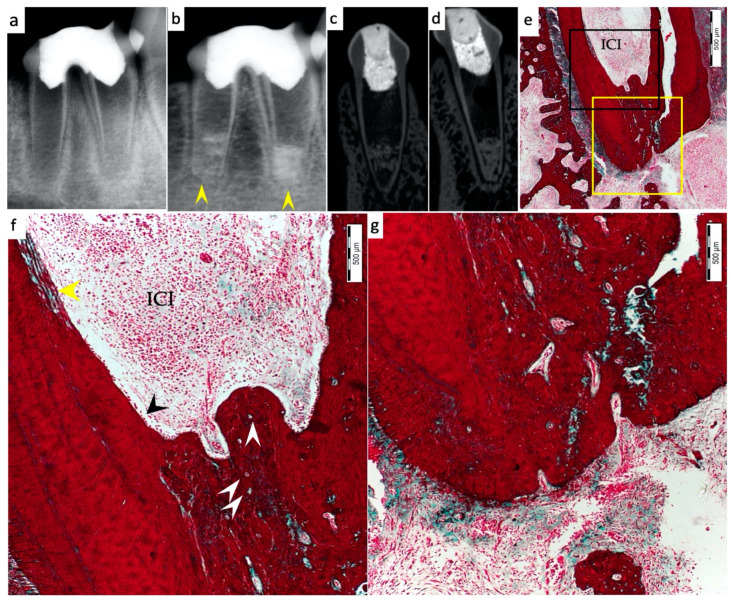
Representative radiographic, µ-CT, and histochemical photomicrographs of root samples from the BC group. (**a**) Immediate postoperative periapical radiograph showing immature roots and (**b**) 13-week postoperative periapical radiograph showing apical closure in both roots (yellow arrowheads). (**c**,**d**) Thirteen-week postoperative µ-CT coronal views of the distal and mesial roots, respectively, with evident apical closure and mineralized tissue deposition in the apical region. (**e**) Histological evidence of the presence of ingrowth deposition of cementum-like hard tissue along the apical region with evident apical closure (MTx200). (**f**) Higher magnification of the black-boxed area in (**e**), showing deposition of cementum-like hard tissue with entrapped cementocytes (white arrowheads) within the apical root hard tissue and lined with cementoblast-like cells (black arrowhead) within the pulp space of the apical region with an evident area of localized, severe chronic inflammation within canal space (ICI); new reparative dentin-like matrix deposition is also apparent along the internal walls of the root canal (yellow arrowhead) (MTx400). (**g**) A magnified, detailed view of the yellow-boxed area in (**e**), showing apical closure of the root with signs of vascularization and periapical inflammation (MTx400).

**Figure 3:**
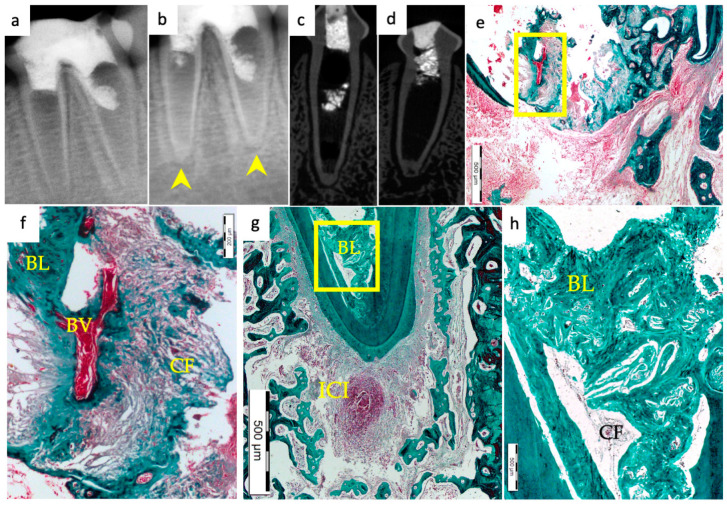
Representative radiographic µ-CT and histochemical photomicrographs of root samples from the BC + HA group. (**a**) Immediate postoperative periapical radiograph and (**b**) 13-week postoperative periapical radiograph confirming apical closure in both roots (yellow arrowheads). (**c**,**d**) µ-CT coronal views of the distal and mesial roots, respectively, show evidence of radiographical apical closure, with apical radiolucency in the mesial root. (**e**) Ingrowth of bone-like tissue within the apical region of the distal root with evident apical closure and dispersed mild inflammatory cells around newly formed hard tissues (MTx200). (**f**) A magnified, detailed view of the yellow-boxed region in (**e**); soft tissue regeneration with blood vessels surrounded by deeply stained collagen fibers (CFs) and the beginning of osteoid bone-like hard tissue formation (BL) (MTx400). (**g**) Formation of new bone-like (BL) hard tissue within the pulp space of the apical region with apical closure and an evident area of localized, severe, chronic periapical inflammation (ICI) (MTx100). (**h**) A magnified detailed view of the yellow-boxed region in (**g**); scattered bone-like tissues are evident, enclosing collagen fibers and surrounding blood vessels with a few dispersed inflammatory cells (MTx400).

**Figure 4:**
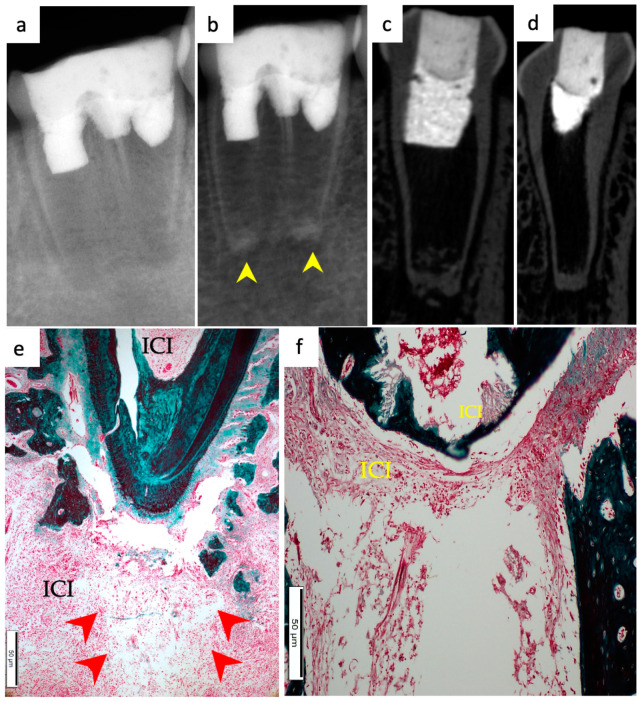
Representative radiographic µ-CT and histochemical photomicrographs of root samples from the BC + HA group. (**a**) Immediate postoperative periapical radiograph and (**b**) 13-week postoperative periapical radiograph confirming apical closure in both roots (yellow arrowheads). (**c**,**d**) Coronal µ-CT views of the mesial and distal roots, respectively, show apical closure and periapical lesions. (**e**) Apical closure of the distal root with moderate periapical and intracanal inflammatory cell infiltrate (ICI) as well as edema within the periapical tissues are apparent (red arrowheads) (MTx100). (**f**) Mesial root showing apical closure with intracanal and periapical accumulation of inflammatory cells (ICI) around an edematous area (MTx200).

**Figure 5:**
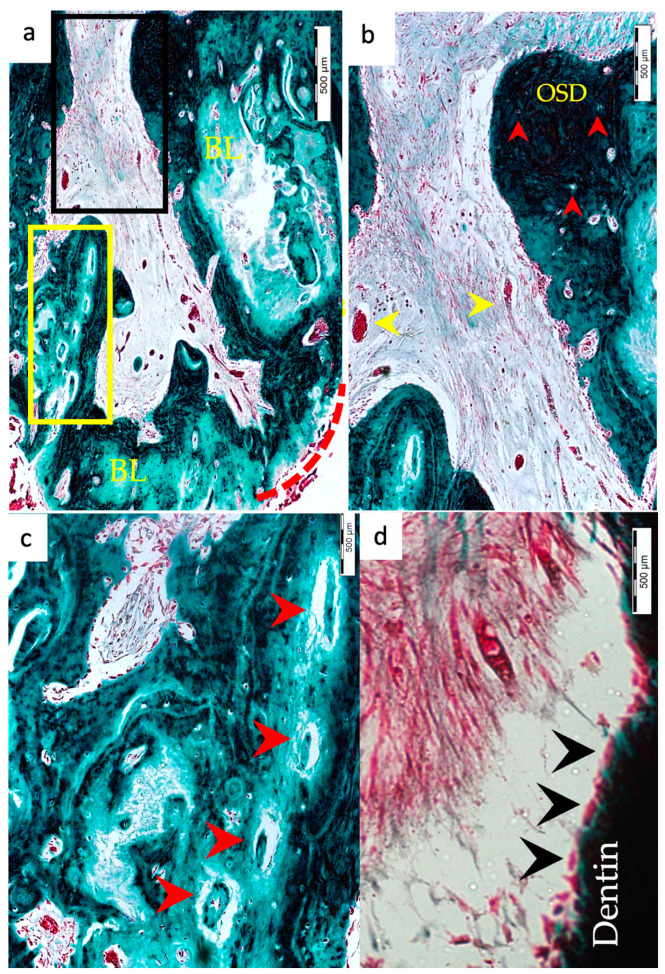
Representative histochemical photomicrographs of root samples from the BC + HA group. (**a**) Formation of a vascularized fibrous connective tissue with dense collagen fibers and the presence of blood vessels and the formation of new bone-like hard tissue (BL) in the apical region with apical closure (red dotted line) (MTX200). (**b**) A magnified, detailed view of the black-boxed region in (**a**); highly vascularized soft connective tissue within the pulp space is evident, including fibroblasts and scattered collagen fibers (CFs), blood vessels (yellow arrowheads), and mild inflammatory cell infiltration with the presence of osteodentin deposition (OSD) showing entrapped osteocyte-like cells (red arrowheads) (MTx400). (**c**) A magnified, detailed view of the yellow-boxed region in (**a**), showing the formation of osteons around blood vessels represented by Haversian canals (red arrowheads) within the bone-like hard tissues (MTX600). (**d**) A detailed magnified view to show reparative dentin wall (black arrowheads); soft connective tissue with dense collagen fibers, fibroblasts, and blood vessels is also seen within the pulp space (MTx1000).

**Figure 6:**
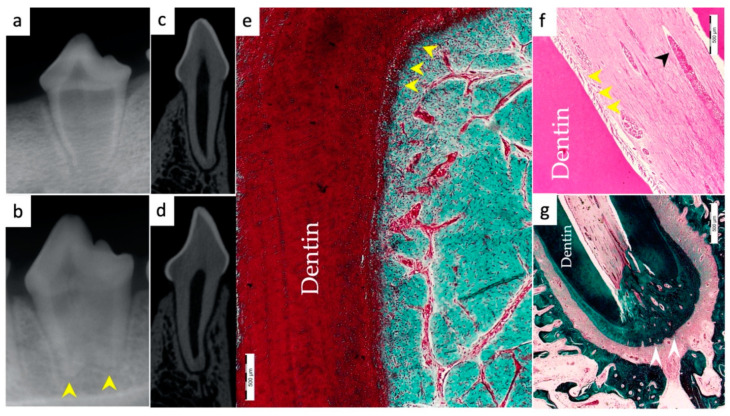
Representative radiographic µ-CT and histological and histochemical photomicrographs of root samples from the positive control group; (**a**) periapical radiograph at the beginning of the experiment and (**b**) 13-week postoperative periapical radiograph confirming apical closure in both roots (yellow arrowheads). (**c**,**d**) Coronal µ-CT views of the mesial and distal roots, respectively, showing apical closure. (**e**) Typical configuration of vital pulp tissue evidenced by odontoblasts lining the predentin and dentin wall (yellow arrowheads); the pulp is densely packed with highly vascularized connective tissue, with the absence of signs of inflammation; a few scattered inflammatory cells among the pulp tissue structures (MTx400). (**f**) Highly vascularized pulp tissue evidenced by odontoblasts lining the dentin wall (yellow arrowheads) and blood vessels (black arrowhead) (H&Ex400). (**g**) Apical closure is evident (white arrowheads), surrounded by dentin and cellular cementum (MTx200).

**Figure 7:**
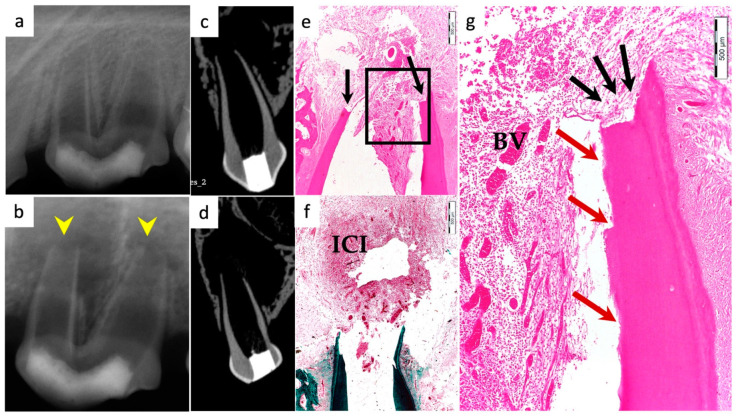
Representative radiographic, µ-CT, and histological and histochemical photomicrographs of root samples from the negative control group. (**a**) Immediate postoperative periapical radiograph and (**b**) 13-week postoperative periapical radiograph showing open apices and periapical lesions related to both roots (yellow arrowheads). (**c**,**d**) Coronal µ-CT views of the mesial and distal roots, respectively, confirm the lack of apical closure and the formation of periapical lesions. (**e**) Absence of any signs of new pulp tissue regeneration with the absence of apical closure (black arrows) (H&Ex200). (**f**) Signs of localized moderate to severe inflammatory cell infiltrates within the apical and periapical region (ICI) surrounded by diffuse and congested blood vessels with the lack of apical closure (MTx200). (**g**) A magnified, detailed view of the black-boxed region in (**e**), showing inflammatory cell infiltrates and congested blood vessels (BV) within the apical region of the canal; lack of apical closure (black arrows) with dentin resorption (red arrows) is also evident (H&Ex400).

Periapical radiographs were taken immediately postoperatively and 13 weeks postoperatively for the experimental groups to demonstrate immature roots and apical closure, respectively, as seen in Figure 1a,b, Figure 2a,b, Figure 3a,b and Figure 4a,b. µ-CT imaging was used to confirm apical closure and the presence or absence of periapical radiolucency for each root, as seen in Figure 1c,d, Figure 2c,d, Figure 3c,d and Figure 4c,d. The resulting numbers and percentages of root samples demonstrating features of the evaluated histological parameters are summarized in Table 2 and presented in Figure 1, Figure 2, Figure 3, Figure 4, Figure 5, Figure 6 and Figure 7.

### 3.1. Hard Tissue Deposition

Histological evidence of hard-tissue deposition on root dentinal walls was seen in 86% of the roots in the BC group (Figure 1 and Figure 2*)* and 80% of the roots in the BC + HA group (Figure 3, Figure 4 and Figure 5*)* (*p* = 0.44).

### 3.2. Type of Hard Tissue Formed

Regarding the type of hard tissues formed within the pulp spaces and apical area of treated roots, cementum-like tissues were the most frequently observed in both groups, compared to other hard tissue types (50% and 45% in the BC and BC + HA groups, respectively). The ingrowth of cementum-like hard tissue deposition with entrapped cementocytes-like cells from root surfaces into the canal walls along the apical area lined with cementoblast-like cells was also observed in both groups (Figure 2e,f).

A newly deposited reparative dentin matrix was also evident in the BC group (22.7% (Figure 1e,f and Figure 2e,f)) and in the BC + HA group (20% (Figure 5b,d)). Osteodentin-like hard tissue was frequently seen deposited on the dentinal root walls, distinguished by the presence of entrapped cells within the darkly stained highly calcified areas (Figure 5b).

The formation of bone-like tissues was also evident in both the BC group (27.3% (Figure 1e,f)) and the BC + HA group (35% (Figure 3g,h and Figure 5a,c)) as scattered hard tissue deposition within the root canal space in the apical region surrounding connective tissue spaces and blood vessels. There were no statistically significant differences regarding the type of hard tissues formed between the two groups (*p* = 0.6).

### 3.3. Vascularization and Formation of Vascularized Soft Connective Tissue

Newly vascularized fibrous connective tissues (CTs) were observed within the root canal spaces surrounding the newly formed hard tissues in 54.6% of the roots in the BC group (Figure 2f) compared to 85% of the roots in the BC + HA group (Figure 3e,f), with a statistically significant difference (*p* = 0.035). However, fibrous connective tissue formation was significantly observed more often in the BC + HA group (40%) (Figure 5a–d) compared to the BC group (9.1%) (*p* = 0.029).

### 3.4. Degree of Inflammation

In the BC group, the majority of the roots (68.2%) showed mild inflammatory cell infiltration entrapped within the regenerated tissues in the pulp space (Figure 1f). At the same time, 18.2% of the roots did not show any inflammation within the canal space or the periapical tissues. The remaining roots (9.1% and 4.5%) showed moderate to severe inflammatory cell infiltration within the pulp space and periapical tissues, respectively (Figure 2e–g). In the BC + HA group, mild inflammation was observed in 45% of the roots, limited to the pulp space (Figure 3e,f and Figure 5b). Meanwhile, moderate inflammation was observed in 35% of the roots (Figure 4c,f). One root from the BC + HA group (10%) showed severe inflammatory cell infiltration in the periapical area despite continued apical closure (Figure 3g). The degree of inflammation was significantly higher in the BC + HA group than in the BC group (*p* = 0.048).

### 3.5. Apical Closure

Histological evidence of apical closure was observed in 72.7% of the roots in the BC group (Figure 1f and Figure 2g) and in 85% of the roots in the BC + HA group (Figure 3g, Figure 4e,f and Figure 5a), which was confirmed using conventional radiographs and µ-CT (Figure 1b–d, Figure 2b–d, Figure 3b–d and Figure 4b–d) in the BC and BC + HA groups, respectively. The apical closure was caused either by a scattered bone-like hard tissue deposition (Figure 1f and Figure 5a) or by the ingrowth of cementum-like hard tissues (Figure 2g). However, these differences were not statistically significant (*p* = 0.46).

### 3.6. Positive and Negative Control Groups

All roots in the positive control group showed evidence of a normal dentin–pulp complex, continued root maturation, and apical closure at the end of the follow-up period (Figure 6a–g). Conversely, in the negative control group, the discontinuation of root maturation was reported in all samples. At the same time, the canal spaces were empty, with remnants of necrotic pulp tissues and the absence of newly formed tissues. Resorption areas in root dentin and cementum were observed. Furthermore, severe inflammatory periapical lesions were evident, and many resorption lacunae were also observed along the apical cementum (Figure 7a–g).

## 4. Discussion

The usefulness and advantages associated with the use of scaffolds in REPs have been demonstrated in previous studies [6,33]. Scaffolds provide a 3D microenvironment for cells to grant them the support required, facilitating their adherence, proliferation, and differentiation and the formation of an extracellular matrix [7]. Hyaluronic acid (HA) scaffolds have been evaluated in multiple in vitro investigations of dental pulp regeneration, and their biocompatibility and potentiality in promoting odontoblastic differentiation of DPSCs were demonstrated [20,21,22,23]. However, FDA-approved dermal fillers commercially available as synthetic HA-based hydrogels offer unique advantages in terms of controlled preparation. They are easy to acquire, handle, and insert into the root canal. These dermal fillers have undergone in vitro assessments in previous investigations [25,26].

Recently, a case report showed promising results when an HA-based injectable hydrogel was used as a scaffold in an immature necrotic central incisor [34]. The present study evaluated the histological outcome of Restylane Lyft when used as a scaffold for dental pulp regeneration in pulpless, non-infected immature teeth in dogs. Infection, the impact of residual bacteria on stem cells [35,36,37], and the cytotoxic effect of disinfecting materials [38,39,40] are confounding variables that can negatively affect the outcomes of REPs. For this purpose, a non-infected model was used in the current study’s design to focus on the solitary effect of Restylane Lyft combined with the blood clot when used as a scaffold in REPs compared to the effect of blood clots alone.

The results presented in the current study showed that the use of Restylane Lyft combined with the blood clot enhanced the formation of intracanal mineralized tissues, which is in accordance with an earlier study [32], where the authors showed that the combination of a scaffold and stem cells gave rise to pulpal regeneration, root lengthening, and the deposition of dentin along the canal walls. The ingrowth of cementum-like tissue from the root surface into the canal walls was frequently seen in most roots from both BC and BC + HA groups in the present study, and bone-like tissue was observed as scattered islands within the canal space at the apical third of the root canals. These findings agree with those reported by previous investigators [41,42,43].

Despite the lack of statistical differences between the experimental groups regarding apical closure, 72% and 85% of the roots showed histological apical closure in the BC and the BC + HA groups, respectively. Roots in the negative control group that were pulpectomized and received no further treatments showed a cessation in root development with the formation of periapical lesions. Consequently, apical closure was lacking.

When combined with the blood clot, Restylane Lyft significantly enhanced vascularization within the pulp spaces and the formation of a vascularized soft connective tissue with the presence of fibroblasts and collagen fibers within the pulp spaces. This could be due to the degradation products of the HA scaffold, supporting the findings of previous researchers, who suggested that the degradation products of low-molecular-weight HA can stimulate endothelial cell proliferation and migration [44,45,46]. Moreover, Restylane Lyft’s longer degradation times allow the scaffolds to maintain their porosity long enough for cells to attach and proliferate [47]. This could be ascribed to the larger particle size of Restylane Lyft, which might affect the scaffold crosslinking density and stability on a dimensional scale [48]. Generally, as the crosslinking density increases, the water content and mass weight decrease and water uptake capacity increases [49]. The water uptake capacity of scaffolds is a desirable trait in tissue engineering because it minimizes the loss of bodily fluid and nutrients from the scaffold during in vitro culture and in vivo implantation; hence, cell adhesion and proliferation are improved [50].

Moderate to severe inflammatory reactions were seen within the pulp space of the treated roots in the present study in both BC and BC + HA groups, with percentages of 13.6% and 45%, respectively. However, compared to the BC group, the BC + HA group showed a significantly higher rate of inflammation. In REPs, inflammation might have a favorable effect on new tissue formation. This was clarified in a study by Wang et al. in 2010. They suggested that the presence of severe inflammation resembles the role of stress in odontoblasts, which triggers the dentinal matrix to deposit reactionary dentin. Thus, inflammation could stimulate the formation of new tissues [43]. However, the significantly higher intensity of inflammation observed in the BC + HA group is worth further exploration. It can also be attributed to the degradation products of the HA scaffold.

It was previously suggested that different forms of degraded materials of HA are capable of binding to cell-surface HA receptor glycoproteins; with such binding, oligomers can congregate on these receptors and thus trigger them to activate associated intracellular inflammatory cascades [51]. In this manner, the released inflammatory cytokines activate the proliferation and migration of non-inflammatory cells, resulting in wound healing or tissue remodeling [52]. This might, in turn, explain the significantly increased formation of a fibrous connective tissue in the BC + HA group compared to the BC group, with percentages of 40% and 9.1%, respectively.

However, this could also be due to the physical properties of the scaffold used. Restylane Lyft, previously known as Perlane, showed medium viscosity (η* = 124,950 cPa) and elasticity (G′ = 541 Pa). These values may also match the properties of the native pulp tissue, which enhances stem cell proliferation and differentiation [49,53]. It was observed that Restylane Lyft played a role in supporting the MTA plug, which maintained its positioning. This may justify the formation of new pulpal tissue in the root canal space of the BC + HA group. Yamauchi et al. [54] previously described the advantages coupled with the use of a scaffold; it aids in applying the MTA plug and hampers its apical migration, thus impeding new tissue formation.

Soft connective tissue within the pulp spaces was evident in some regenerated areas near the apical region with the coexistence of fibroblasts, collagen fibers, and blood vessels. In fact, it has been well documented in various studies that HA exhibits a fibroblast enhancement property. Its application at wound sites improved healing via the increased activity of fibroblasts and the production of collagen fibers and elastin [55,56,57,58,59]. In contradistinction to the presented results, in previously reported histological studies that tested different scaffolds while using infected models, the formation of pulp-like connective tissue was lacking, suggesting that stem cells from the apical papilla might have undergone necrosis as a result of induction from periapical infection and/or the application of a triple antibiotic paste used for canals’ disinfection [32,43,54,60].

Limitations in the present study mainly arise from the short time elapsed until euthanasia. Restylane Lyft is known to have prolonged degradation times. Therefore, more extended observation periods are needed to monitor the effect of the degradation process and the resulting degree of inflammation over time to better understand the underlying mechanism of the formation and regeneration of the soft connective tissue within the pulp spaces. Moreover, further investigations are necessary to evaluate the effect of the Restylane Lyft scaffold alone, without the blood clot. Finally, other known affecting factors (i.e., infection and disinfection) need to be considered in designing future studies.

## 5. Conclusions

The results of the present study suggest that Restylane Lyft combined with a blood clot used as a scaffold may improve the outcomes of REPs in non-infected, pulpless, immature teeth in dogs and is worth further exploration.

## Figures and Tables

**Table 1:** Histological evaluation criteria.

(1) Histological hard tissue deposition on radicular dentin walls	Present Absent
(2) Type of hard tissues formed within the root canal space	Absent Dentin-like matrix: Presence of odontoblast-like cells with associated odontoblastic processes, predentin, and/or dentin-like calcified areas Bone-like: Presence of a Haversian system with uniformly distributed osteocyte-like cells Cementum-like: Cementum-like mineralized tissue lining and adhering onto the dentin of the root with or without embedded cementocyte-like cells
(3) Vascularization	Present Absent
(4) Presence or absence of soft connective tissues within root canal space	Present (new vascularized fibrous connective tissue with fibroblasts and collagen fibers) Absent
(5) Degree of inflammatory cells infiltrated within pulpal canal space and/or in periapical area	None (0): Absence of inflammatory cells Mild (1): Small number of dispersed inflammatory cells Moderate (2): Focal aggregation of inflammatory cells Severe (3): Intense inflammatory infiltrate and tissue alterations
(6) Presence or absence of histological apical closure	Present Absent

**Table 2:** Histological findings of the experimental groups after 13 weeks.

Parameter	NC (*n* = 10)	PC (*n* = 11)	BC (*n* = 22)	BC + HA (*n* = 20)	*p*-Value
(1) Histological hard tissue deposition on radicular dentin walls					
Present	0% (0)	100% (11)	86.3% (19)	80% (16)	0.44 *
Absent	100% (10)	0% (0)	13.4% (3)	20% (4)	
(2) Type of hard tissues formed along the root canal walls and within pulp spaces					
Absent			0% (0)	0% (0)	0.6 *
Dentin-like			22.7% (5)	20% (4)	
Bone-like			27.3% (6)	35% (7)	
Cementum-like			50% (11)	45% (9)	
(3) Vascularization					
Present	0% (0)	100% (11)	54.6% (11)	85% (17)	0.04 *
Absent	100% (10)	0% (0)	45% (9)	15% (3)	
(4) Presence or absence of vascularized soft connective tissues within root canal space					
Present	0% (0)	100% (11)	9.1% (2)	40% (8)	0.029 *
Absent	100% (10)	0% (0)	90.9% (20)	60% (12)	
(5) Inflammation within pulp spaces and/or the periapical area					
None	0% (0)	81.8% (9)	18.2% (4)	10% (2)	0.048 †
Mild	0% (0)	18.2% (2)	68.2% (15)	45% (9)	
Moderate	10% (1)	0% (0)	9.1% (2)	35% (7)	
Severe	90% (9)	0% (0)	4.5% (1)	10% (2)	
(6) Apical closure					
Present	0% (0)	100% (11)	72.7% (16)	85% (17)	0.46 *
Absent	100% (10)	0% (0)	27.3% (6)	15% (3)	

* Fisher exact test. † Mann–Whitney U test. The *p*-value column represents comparisons between BC and HA groups, where *p* ≤ 0.05. NC: negative control, PC: positive control, BC: blood clot, and HA: Restylane Lyft.

## Data Availability

The data presented in this study are available on request from the corresponding author. The data are not publicly available due to copyright.
